# A Portable Dual-Mode Microfluidic Device Integrating RT-qPCR and RT-LAMP for Rapid Nucleic Acid Detection in Point-of-Care Testing

**DOI:** 10.3390/bios16010051

**Published:** 2026-01-08

**Authors:** Baihui Zhang, Xiao Li, Mengjie Huang, Maojie Jiang, Leilei Du, Peng Yin, Xuan Fang, Xiangyu Jiang, Feihu Qi, Yanna Lin, Fuqiang Ma

**Affiliations:** 1 Shandong Lab of Advanced Biomaterials and Medical Devices in Weihai, Weihai 264210, China; 2 Medical Enzyme Engineering Center, CAS Key Lab of Bio-Medical Diagnostics, Suzhou Institute of Biomedical Engineering and Technology, Chinese Academy of Sciences, Suzhou 215613, China; 3 Weihai Huakang Medical Laboratory Co., Ltd., Weihai 264210, China; 4 Suzhou Ker Life Technology Co., Ltd., Suzhou 215163, China

**Keywords:** POCT, RT-LAMP, RT-qPCR, TV-qPCR, Tesla valves

## Abstract

Point-of-care testing (POCT) has emerged as a vital diagnostic approach in emergency medicine, primary care, and resource-limited environments because of its convenience, affordability, and capacity to provide immediate results. Here, we present a multifunctional portable nucleic acid detection platform integrating reverse transcription polymerase chain reaction (RT-qPCR) and reverse transcription loop-mediated isothermal amplification (RT-LAMP) within a unified microfluidic device. The system leverages Tesla-valve-based passive flow control to enhance reaction efficiency and operational simplicity. A four-channel optical detection unit allows for multiplex fluorescence quantification (CY5, FAM, VIC, ROX) and has high sensitivity and reproducibility for RT-LAMP. The compact design reduces the overall size by approximately 90% compared with conventional qPCR instruments. For RT-PCR, the system achieves a detection limit of 2.0 copies μL^−1^ and improves analytical efficiency by 27%. For RT-LAMP, the detection limit reaches 2.95 copies μL^−1^ with a 14% enhancement in analytical efficiency. Compared with commercial qPCR instruments, the device maintains equivalent quantitative accuracy despite significant miniaturization, ensuring reliable performance in decentralized testing. Furthermore, the total RT-LAMP assay time is reduced from more than two hours to 42 min, enabling truly rapid molecular diagnostics. This dual-mode platform offers a flexible, scalable strategy for bridging laboratory-grade molecular assays with real-time POCT applications, supporting early disease detection and epidemic surveillance.

## 1. Introduction

Point-of-Care Testing (POCT) is a modern diagnostic approach characterized by core features such as ease of use, cost-effectiveness, environmental compatibility, and independence from specialized laboratory personnel [1,2]. By enabling rapid on-site analysis, POCT delivers immediate results directly to clinical decision-makers. This significantly reduces the delays associated with sample transportation and preprocessing, making it particularly suitable for emergency settings, primary care, and resource-limited environments [3,4]. Various technologies, including microfluidic chips, lateral flow chromatography strips, and biosensors, are employed to achieve “near-patient” testing. This proximity shortens the diagnostic cycle and accelerates therapeutic interventions [5,6]. While reverse transcription polymerase chain reaction (RT-qPCR) remains the gold standard for viral detection due to its high sensitivity and specificity—particularly valuable in laboratory settings requiring precise quantitative analysis, such as viral load monitoring and mutant strain identification [7,8]—it relies on thermocycling-based amplification and sophisticated instrumentation. Although capable of achieving detection limits as low as single-copy RNA, the RT-qPCR assay typically requires 2–4 h [8,9]. In contrast, Reverse Transcription Loop-mediated Isothermal Amplification (RT-LAMP) operates via an isothermal mechanism, eliminating the need for thermal cycling equipment. RT-LAMP requires only a constant temperature source and can complete amplification within 15–60 min. These attributes give RT-LAMP greater potential for application in bedside testing and resource-limited areas [10,11].

Advances in POCT devices in recent years have focused on three major directions: sensitivity enhancement, miniaturization and automation, and precise quantification and high-throughput detection. Sensitivity is a critical parameter for POCT devices. Jayanath et al. (2018) pioneered the development of an Hepatitis B Virus (HBV) Deoxyribonucleic Acid (DNA) detection system integrating LAMP with electrochemical sensing [12]. This system synchronized the amplification and detection steps by monitoring the reaction in real time using redox indicators [12]. Fei Hu et al. (2019) innovatively introduced immiscible filtration assisted by surface tension (IFAST) technology, combined with microdroplet digital detection and smartphone fluorescence imaging [13]. This approach significantly improved the detection sensitivity for low-concentration DNA (achieving a detection limit of 10 copies/μL), thereby overcoming a key technical bottleneck of traditional POCT equipment [13]. By 2021, Chen et al. successfully realized the simultaneous detection of multiple Severe Acute Respiratory Syndrome Coronavirus 2 (SARS-CoV-2) targets by combining multiplex reverse transcription LAMP (mRT-LAMP) with a nanoparticle lateral flow biosensor (LFB) [14]. Their system completed the entire process, from Ribonucleic Acid (RNA) extraction to result interpretation, within 80 min, representing a 60% increase in efficiency compared to conventional qPCR [14]. Miniaturization and automation determine the applicable scenarios of POCT devices. Srivastava et al. (2021) developed a paper-based LAMP detection device enabling rapid screening of pathogen nucleic acids without specialized equipment through colorimetric visualization [15,16]. Its modular design facilitates the detection of different targets by primer substitution [15,16]. Kang et al. (2021) made a breakthrough by adopting the nanoplasmonic photothermal effect to construct an on-chip self-feeding qPCR device [17]. This innovation compressed the time required for 40 amplification cycles to 306 s, nearly 20-fold faster than traditional thermal cyclers [17]. In 2022, Nguyen et al. developed an IoT-integrated microfluidic system [18]. By integrating sample lysis, nucleic acid amplification, and real-time monitoring modules within the chip, combined with smartphone data transmission, they achieved the first fully automated and remotely monitored testing process [18]. Precise quantification and high-throughput testing represent the threshold from qualitative testing to precision medicine. Hoan et al. (2022) developed a 3D-printed plasma isolation-RT-qPCR system capable of accurately quantifying Human Immunodeficiency Virus (HIV) viral load in whole blood samples [19]. Its power-free design significantly expands the application scenarios in resource-restricted areas [19]. Liu et al. (2023) developed a fingertip blood HIV testing device that innovatively integrates semi-quantitative RT-LAMP with smartphone image analysis [20]. Achieving an 86% viral recovery rate and 95% clinical grading accuracy, it provides decision support for individualized treatment [20]. Zhang et al. (2023) introduced a Lab-on-Optical-Disc (LOAD) system that integrates droplet generation, PCR amplification, and data analysis [21].

Commercially available viral testing POCT devices can be broadly categorized into kit-based and instrument-based types. While most RT-LAMP kits are compact, they are typically designed for single-virus detection, limiting their versatility and user convenience for carrying and use. Conversely, instrument-based POCT devices, which offer broader applicability, still have room for improvement regarding detection limits and assay time. Although real-time reverse transcription polymerase chain reaction (RT-qPCR)-based methods offer high sensitivity and quantitative accuracy, they usually require bulky and expensive instruments, presenting challenges for POCT applications. On the other hand, methods based on isothermal amplification techniques can utilize lower-cost instruments but generally provide only semi-quantitative results, indicating limited quantitative accuracy. Overall, viral POCT devices are progressing towards greater portability and efficiency. However, no device currently on the market successfully integrates ‘small size’, ‘high efficiency’, ‘low detection limits’, and ‘multi-channel’ capabilities. This gap persists due to the stringent primer quality requirements of LAMP amplification (increasing susceptibility to false positives) and the inherent bulkiness and temperature dependency of conventional qPCR equipment, which hinder portability. To address these limitations, we propose a multi-channel, efficient, and user-friendly POCT device that integrates LAMP and qPCR methodologies into a single platform.

In this study, we developed a portable, multifunctional nucleic acid detection device (TV-qPCR) integrating both RT-qPCR and RT-LAMP technologies, providing a flexible solution for rapid POCT. By designing a specialized fluorescence detection chip and optimizing the Tesla valve structure, we successfully prevented liquid backflow in microfluidic channels. The system incorporates a meticulously designed four-channel optical detection system capable of simultaneously quantifying the fluorophores Cy5, 6-carboxyfluorescein (FAM), violet invader (VIC) and rhodamine X (ROX) during a single sample run. Moreover, the device achieves a 90% volume reduction compared to conventional systems while requiring smaller sample volumes. Under identical amplification conditions, the TV-qPCR system attains a detection limit of 2.0 copies/μL in RT-qPCR, demonstrating 27% higher single-sample detection efficiency than traditional qPCR instruments. The detection limit (LOD) for TV-qPCR using RT-LAMP is 2.95 copies/μL, which is 23% lower than that of traditional qPCR (3.64 copies/μL). The improvement in quantification limit (LOQ) is more significant: TV-qPCR (26.47 copies/μL) shows a 47% reduction compared to control systems (50.09 copies/μL). Relative to traditional RT-qPCR, which requires over 2 h per detection cycle, TV-qPCR enables complete RT-LAMP processing—including sample lysis, nucleic acid amplification, and result interpretation—within 42 min.

**Figure 1:**
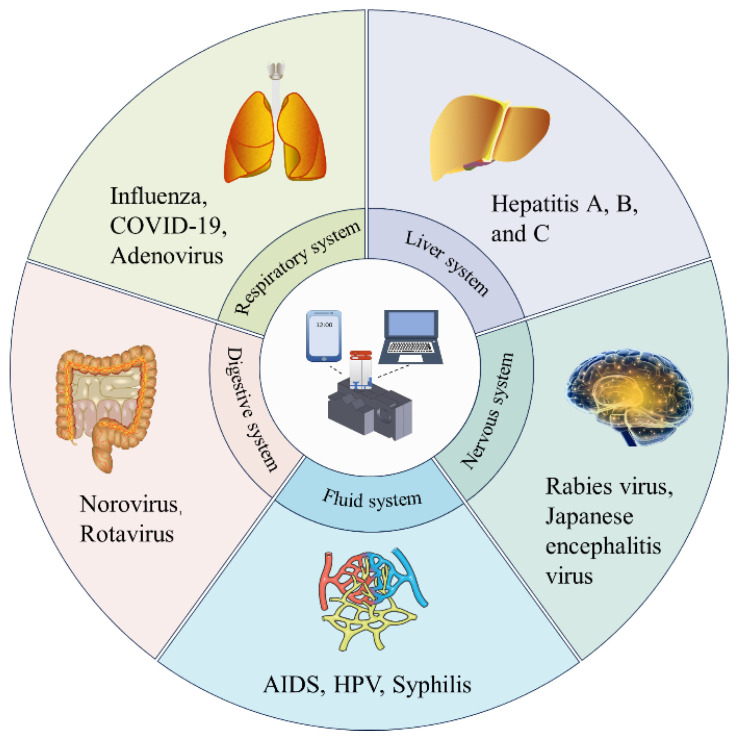
TV-qPCR equipment application scenarios. TV-qPCR equipment can detect a variety of viruses by changing templates and primers, which is suitable for screening different diseases.

This system addresses the urgent need for rapid testing in primary healthcare settings while maintaining laboratory-grade diagnostic accuracy, making it particularly suitable for implementing hierarchical diagnosis and treatment protocols in complex scenarios such as field hospitals and port quarantine stations (Figure 1).

## 2. Materials and Methods

### 2.1. Reagents

Brands of qPCR instruments: real-time fluorescence quantitative PCR instrument (FQD-96A) from Hangzhou BORI Technology Co., Ltd., Hangzhou, China; novel coronavirus (2019-nCoV) *ORF1ab*, *N* and *E* genes, as well as Influenza A H1N1 and Influenza B Victoria were obtained from Guangzhou Bundesheng Bio-Technology Co, Guangzhou, China. Mix reagents were obtained from Suzhou Jin Yizhen Biotechnology Co., Ltd., Suzhou, China of which the lyophilised reagent bulbs contained fluorescent markers, polymerase, four deoxyribonucleoside triphosphate (dNTPs), and potential of hydrogen (PH) buffer ions; the qPCR primer sequences and the primers for LAMP amplification of the *N* gene of the new coronavirus are shown in Tables S1 and S2. Clinical samples were obtained from the Sixth People’s Hospital of Nantong City, Jiangsu Province, China. RT-qPCR kit: 2xHS-RT-PCR Mix (-Ab) is a one-step premix containing all necessary components, including hot-start (antibody-modified) Taq DNA polymerase, reverse transcriptase, dNTPs, an optimized buffer system, and stabilizers. Amplification requires only the addition of primers and template. (Catalog No.: P0008, Manufacturer: Suzhou Jinyizhen Biotechnology Co., Ltd., Suzhou, China). RT-LAMP reagent: RT-SLAMP Mix (SYBR+) Lyophilised Beads is a one-step lyophilised bead formulation containing Bst-like isothermal DNA polymerase, reverse transcriptase, a fluorescent reporting chemistry (SYBR Green dye), and a highly stable reaction buffer. (Catalog No.: D0033, Manufacturer: Suzhou Jinyizhen Biotechnology Co., Ltd., China).

### 2.2. TV-qPCR Instrument Performance Test Method

This study employed a commercial real-time fluorescence quantitative PCR instrument (FQD-96A; Hangzhou Bioer Technology, Hangzhou, China) for parallel comparative experiments with our self-developed TV-qPCR system. Pseudovirus standards containing SARS-CoV-2 and influenza A/B (Flu A/B) gene sequences served as test targets. Key performance metrics evaluated included linear range, multi-channel detection capability, sensitivity, amplification efficiency, system stability, and detection precision. Gradient-diluted pseudovirus samples were prepared in 3–5 replicates and analyzed following standard operating procedures. All samples underwent simultaneous testing using conventional qPCR instruments, whereas TV-qPCR’s physical structure permitted only single-sample processing per run.

#### 2.2.1. Sensitivity: Limit of Detection (LOD) and Limit of Quantification (LOQ)

##### TV-qPCR System Operation Procedure

The integrated microfluidic chip was used to complete sample processing and amplification. After connecting the vacuum chamber to the chip, 15 μL of pseudovirus sample (1 μL), forward and reverse primers (0.3 μL each), fluorescent probe (0.3 μL), premix (8.1 μL of 2× Probe Master Mix), lysis buffer, and water were loaded into the sample chamber. The dual-chamber pressure switch was then activated to simultaneously achieve virus lysis and liquid mixing. Following synchronous lysis and mixing, the mixture was precisely distributed to the reaction chamber through the Tesla-valve-controlled microchannel. The reaction program is set as follows: preheat the instrument for 30 min; reverse transcription at 50 °C for 15 min; pre-denaturation at 95 °C for 2 min; followed by 45 cycles of 95 °C for 10 s, 60 °C for 30 s. Real-time fluorescence data were collected at the conclusion of each 60 °C annealing/extension step. The SARS-CoV-2 samples had dilution gradients of 6.0 × 10^2^, 6.0 × 10^3^, 6.0 × 10^4^, 1.2 × 10^5^, and 6.0 × 10^5^ (copies/µL); influenza A virus samples ranged from 9.52 to 9.52 × 10^4^ (copies/µL); and influenza B virus samples ranged from 3.89 × 10^1^ to 3.89 × 10^5^ (copies/µL). Light protection was maintained throughout chip processing to ensure photosensitive dye stability, and thermal cycling commenced immediately upon chip loading using parameters identical to the conventional system.

##### TV-LAMP System Operation Procedure

A 15 μL LAMP reaction system was prepared as follows: Pseudovirus sample template (1 μL) and specific primers (FIP/BIP: 0.16 μL each; F3/B3: 0.02 μL each; LF/LB: 0.04 μL each) were dissolved in nuclease-free water alongside pre-loaded lyophilized reagent spheres in the chip inlet. Vacuum pressure drove dissolution and transport of reagents to the reaction chamber. The amplification protocol comprised 40 cycles of 60 °C for 1 min with heated lid at 105 °C. SARS-CoV-2 *N* gene samples used five dilution gradients: 3.52 × 10^3^, 1.76 × 10^4^, 8.8 × 10^4^, 4.4 × 10^5^, and 2.2 × 10^6^ copies/μL. Fluorescence signals were monitored in real time throughout. All chip handling occurred under light-protected conditions to maintain photosensitive dye stability, with immediate program initiation post-loading (parameters identical to conventional systems). Data analysis followed completion of all replicate experiments.

##### Ct Value Determination Method

The text will describe that amplification curves were generated from the fluorescence readings, and the Cycle threshold (Ct) values were automatically determined by the instrument’s software using the baseline/threshold method. A brief explanation will be added: the baseline was set within the early cycles where no significant fluorescence increase was detected, and the threshold was automatically set at a level significantly above the baseline fluorescence to distinguish specific amplification signal from background noise across all samples in the run.

#### 2.2.2. Linearity/Amplification Curve/Stability/Reproducibility and Precision/Efficiency

The experimental procedure is described in Section 2.2.1, and data were analyzed at the end of all replicated experiments.

### 2.3. Statistical Analyses

Data were statistically analyzed by Excel software and results are expressed as mean ± standard deviation (SD). All experimental techniques are repeated three times or more, and three representative sets of results are selected for presentation. Perform linear regression analysis using an Excel spreadsheet and calculate the R-value of the horizontal and vertical coordinate values based on the formula. Using statistical methods to calculate LOD/LOQ through calibration curves, a series of low concentration samples were taken to measure response values, and the standard deviation (σ) of the regression line in the low concentration region was calculated. According to the ICH guidelines, the recommended formula is LOD ≈ 3.3 × σ/slope and LOQ ≈ 10 × σ/slope. The slope is the slope of the calibration curve, and σ is the standard deviation of the response signal when approaching the LOD level. This quantitative method essentially corresponds to the concepts of signal-to-noise ratios of 3 and 10.

## 3. Results

### 3.1. The Principle of TV-qPCR

**Figure 2:**
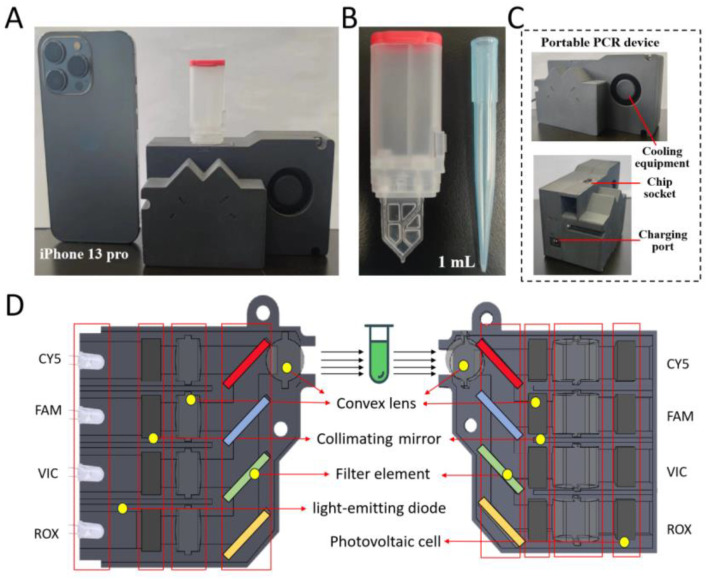
TV-qPCR Equipment Introduction Diagram. (**A**), The system mainly consists of TV-qPCR device, detection chip, the overall volume compared with mobile phone as shown in the figure. (**B**), The chip mainly consists of vacuum chamber and Tesla chip, the size is close to the 1 mL tip. (**C**), Description of the components and interfaces of the TV-qPCR instrument. (**D**), Distribution of the internal components of the TV-qPCR instrument and the description.

We developed a compact POCT molecular detection device (Figure 2A) equipped with a customized microfluidic detection chip (Figure 2B). The chip integrates two functional components: a vacuum chamber and a Tesla-valve-based microfluidic unit (Figure S1A). After the reaction mixture is introduced into the sample-loading chamber, rotating the vacuum chamber switch generates negative pressure that automatically drives the liquid sample into the reaction chamber through the passive Tesla valve (Figure S1B). This pump-free design enables autonomous fluid transfer and effectively prevents sample backflow, offering an efficient and contamination-resistant solution for on-site molecular diagnostics.

The TV-qPCR system comprises three major subsystems: a temperature control module, an optical detection module, and a light source module (Figure 2C). The temperature control unit integrates a thin-film microheater and compact cooling components, achieving rapid thermal cycling for nucleic acid amplification with high precision. The optical detection system is configured with four fluorescence channels (CY5, FAM, VIC, and ROX), enabling simultaneous multiplexed detection (Figure 2D).

Each optical channel is equipped with a dedicated light-emitting diode (LED), collimating mirror, convex lens, and filter element in the excitation pathway, while the emission pathway includes matched photodiodes, collimating optics, and dichroic mirrors. The optical layout positions CY5 (the longest wavelength channel) at the top, followed by FAM, VIC, and ROX in ascending order of wavelength, thereby minimizing spectral overlap and maximizing fluorescence detection efficiency.

Overall, the system is comparable in size to a smartphone and is equipped with a 72 W power adapter (Figure S2), integrating precise temperature regulation, efficient passive fluid control, and multi-channel optical detection into a compact platform. This integration enables rapid, accurate, and fully automated nucleic acid analysis, demonstrating strong potential for point-of-care deployment and resource-constrained molecular diagnostics.

### 3.2. The Principle of Tesla-Valve-Based Chip

**Figure 3:**
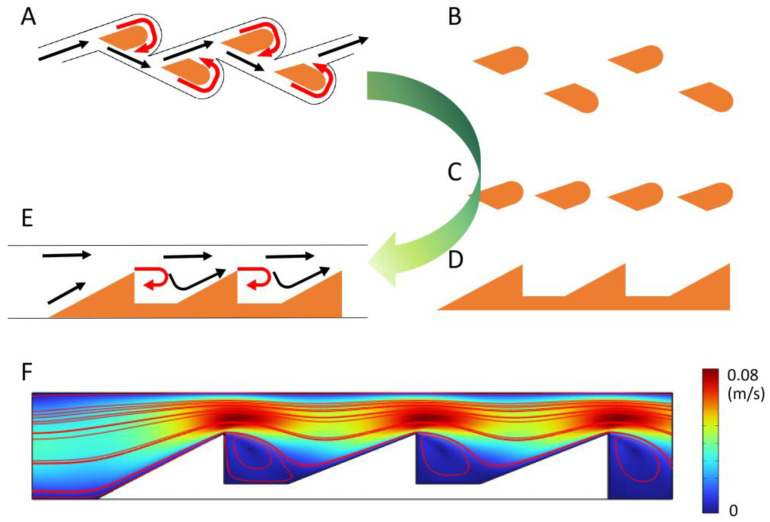
The design and optimization of Tesla valves. (**A**–**E**) depict the structural evolution of the Tesla valve from traditional to simplified designs. (**F**) presents a simulation model of the novel Tesla valve, confirming its one-way flow capability. The black arrow indicates the normal direction of liquid flow, while the red arrow indicates the direction of liquid backflow.

The Tesla valve is a widely used passive check valve that prevents liquid backflow through a geometrically induced flow resistance (Figure 3A). In this study, by optimizing the Tesla valve structure, we transformed the typical two-side swirl channel mode into a one-side swirl channel mode, thereby simplifying the internal geometry and reducing flow resistance. This modified design is particularly suitable for liquid flow with high viscosity in microscale channels, where excessive structural complexity can otherwise amplify viscous losses. The evolved configuration maintains the passive rectification behavior of the Tesla valve while improving manufacturability and integration within microfluidic chips.

To validate the backflow-suppression performance of the optimized design, we performed computational simulations using COMSOL Multiphysics v6.2 (Figure 3F). The fluid–solid coupling was modeled with the liquid region defined as a deformation domain. The fluid dynamics follow the incompressible Navier–Stokes equations for the velocity field u (u, v) and pressure p within a spatially deformed coordinate system:

$$\rho \frac{\partial u}{\partial t}-\nabla \times [-pI+\eta (\nabla u+{\left(\nabla u\right)}^{T})]+\rho (\left(u-u_{m}\right)\times \nabla )u=F$$

−∇×u=0

where I is the unit diagonal matrix, and F is the volumetric force acting on the fluid. In this model, gravitational and other volumetric forces were neglected (F = 0). The inlet boundary (left side) was defined as a fully developed laminar flow with a parabolic velocity profile whose magnitude varies with time according to:

$$u_{in}=\frac{U{\;\times \;t}^{2}}{\sqrt{{\left(0.04-t^{2}\right)}^{2}+{\left(0.1t\right)}^{2}}}$$

where *t* is time (s) and U is the steady-state velocity amplitude. At the outlet (right boundary), pressure was set to *p* = 0. No-slip boundary conditions (u = v = 0) were applied on solid walls, while the deformed interface velocity matched the rate of deformation (u_0_ = u_*t*, v_0_ = v_*t*).

As shown in Figure 3F, distinct recirculating vortices form behind each triangular obstacle, which dissipate reverse kinetic energy and eliminate backflow. The formation of these stable vortices effectively converts part of the flow energy into localized circulation zones, reducing reverse flow even under fluctuating inlet pressures. This mechanism demonstrates the passive rectification ability of the structure without the need for external actuation.

Compared to active microvalves, which require pneumatic, magnetic, or electrical control systems, our optimized passive design operates entirely autonomously, thereby minimizing fabrication complexity and power consumption. Unlike capillary-based valves, whose performance is highly sensitive to surface properties and sample composition, the proposed design maintains a stable flow rectification ratio even for high-viscosity liquids (up to 100 mPa·s). Thus, the modified Tesla valve chip effectively addresses the key challenge of backflow in viscous microflows, ensuring unidirectional, stable, and contamination-free operation for lab-on-a-chip applications.

### 3.3. Performance Validation of POCT Devices

#### 3.3.1. Sensitivity Testing

Sensitivity, as a core performance indicator for detection instruments, reflects a device’s minimum detectable concentration of target analytes. In this study, we systematically evaluated the sensitivity of the TV-qPCR system using SARS-CoV-2 genes (Open Reading Frame 1ab *ORF1ab*, Nucleocapsid Protein *N*, Envelope Protein *E*) and influenza viruses (influenza A IFA, influenza B IFB) as detection targets, with a mainstream commercial instrument (BIO-RAD CFX96 Real-Time qPCR System) as the control (Figure S3).

**Figure 4:**
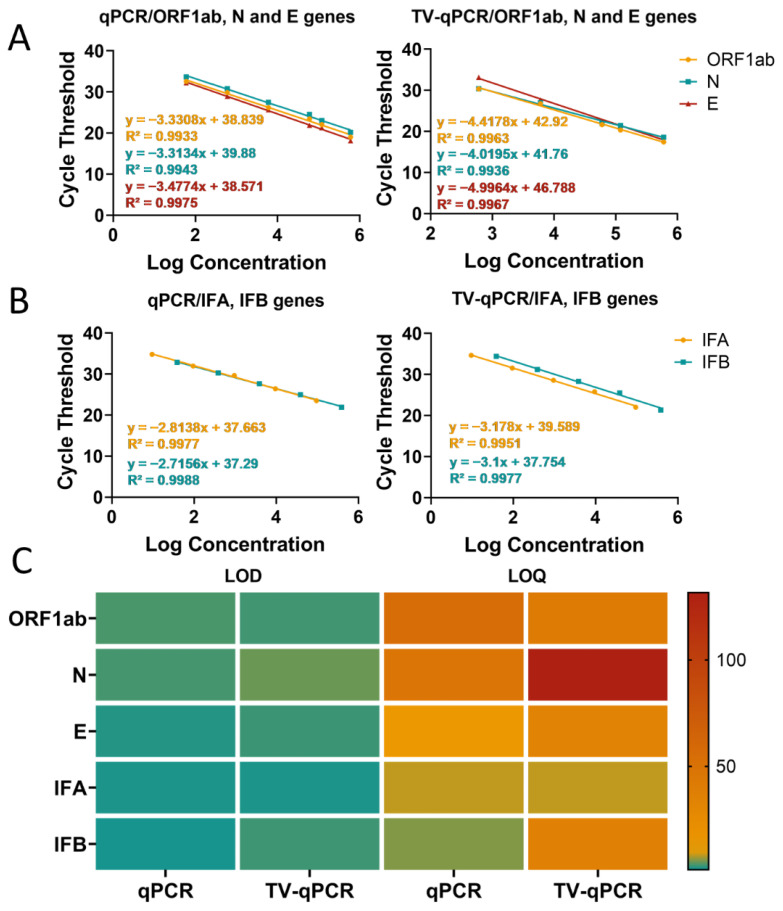
Linearity range and sensitivity testing of the portable multifunctional nucleic acid detection device (TV-qPCR) versus conventional fluorescence quantitative nucleic acid detection equipment (qPCR) for reverse transcription polymerase chain reaction (RT-qPCR). (**A**), Linear detection range of novel coronavirus genes *ORF1ab*, *N*, and *E* by TV-qPCR device and qPCR device. (**B**), Linear detection range of IFA and IFB by TV-qPCR device and qPCR device. (**C**), LOD value, LOQ value of novel coronavirus genes *ORF1ab*, *N*, and *E* and IFA and IFB by TV-qPCR device and qPCR device, and IFB detection with LOD values, LOQ values.

Experimental data revealed differential TV-qPCR performance across targets. For SARS-CoV-2 genes (*ORF1ab*, *N*, *E*), TV-qPCR demonstrated a linear detection range of 6.0 × 10^2^–6.0 × 10^5^ copies/μL, achieving the lower detection limit by one order of magnitude compared to the control qPCR instrument (6.0 × 10^1^–6.0 × 10^5^ copies/μL) (Figure 4A). For influenza A/B virus detection, both systems showed comparable linear ranges (9.5–9.5 × 10^4^ copies/μL) (Figure 4B). Notably, while TV-qPCR detected targets in very low-concentration samples (<10^2^ copies/μL), quantitative reliability may be reduced at these levels, suggesting these results should be interpreted qualitatively.

Comparative analysis of detection limits (LOD) and quantification limits (LOQ) (Figure 4C) further demonstrated that for SARS-CoV-2 targets, TV-qPCR exhibited slightly higher LOD (3.17 copies/μL) and LOQ (33.08 copies/μL) values than the control qPCR system (LOD 2.29 copies/μL, LOQ 12.33 copies/μL). For influenza virus detection, TV-qPCR likewise showed marginally higher LOD (2.00 copies/μL) and LOQ (8.21 copies/μL) compared to the control instrument (LOD 1.79 copies/μL, LOQ 5.87 copies/μL). Despite these modest sensitivity differences (<1.5-fold variation), TV-qPCR achieves significant miniaturization advantages—delivering LOD performance within one order of magnitude while operating at tenfold lower volumes than conventional systems. This demonstrates an optimal engineering trade-off between portability and analytical sensitivity.

Comprehensive evaluation confirms the TV-qPCR system achieves detection sensitivity comparable to conventional qPCR instruments within a miniaturized footprint (~90% size reduction). This balanced technological approach provides unique advantages for POCT and field screening applications, offering a novel solution for rapid pathogen detection.

#### 3.3.2. Reaction Efficiency Test

Detection efficiency, a core performance indicator for molecular diagnostics, is critically significant in POCT scenarios—particularly when addressing emerging infectious diseases where improved timeliness directly shortens epidemic response windows. Our study revealed that under identical amplification protocols, the TV-qPCR system processes individual samples 27% faster than conventional qPCR instruments (Table S3). This technological breakthrough originates from two key innovations.

First, TV-qPCR achieves cap-free thermal operation through its integrated Tesla valve microfluidic chip. While traditional qPCR requires heated lids (typically 105 °C) to prevent evaporation, TV-qPCR employs a bidirectional hydrodynamic control system: low-pressure adsorption accelerates directional fluid transport during forward flow, while vortex deceleration establishes dynamic equilibrium during reverse flow. This hydrodynamic regulation effectively prevents liquid splashing and evaporation, eliminating lid heating requirements and reducing processing time by approximately 15% per run.

The engineering realization of this efficiency advantage has enabled the TV-qPCR system to demonstrate its unique value in scenarios such as on-site screening for outbreaks and rapid diagnosis in primary care settings.

#### 3.3.3. Stability Testing

**Figure 5:**
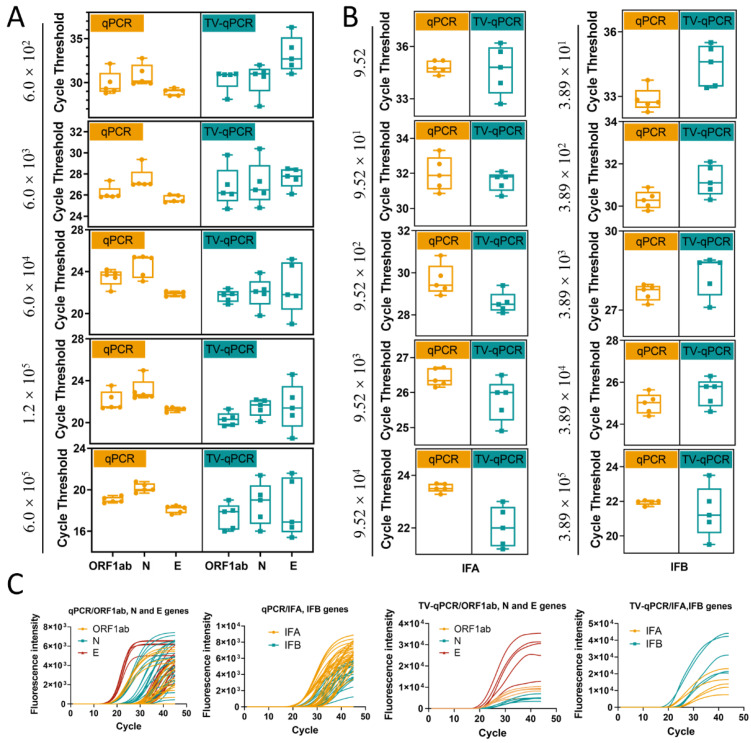
Stability and amplification curve testing of portable multifunctional nucleic acid detection devices (TV-qPCR) versus conventional fluorescence quantitative polymerase chain reaction (qPCR) equipment for reverse transcription-polymerase chain reaction (RT-qPCR). (**A**), Data stability of the TV-qPCR device versus the qPCR device for the novel coronavirus genes *ORF1ab*, *N*, and *E*. (**B**), Data stability of the TV-qPCR device versus the qPCR device for the influenza A IFA and influenza B IFB assays. (**C**), Amplification curves of the TV-qPCR device versus the qPCR device for the novel coronavirus genes *ORF1ab*, *N*, and *E* as well as the amplification profiles for influenza A IFA and influenza B IFB detection.

Equipment stability is fundamental for clinical testing reliability, directly impacting result reproducibility and traceability. To evaluate TV-qPCR’s operational reliability in real-world scenarios, we systematically assessed data stability through multiple replicate experiments (Figure 5). Data analysis revealed: for SARS-CoV-2 genes (*ORF1ab*, *N*, *E*), traditional qPCR exhibited cycle threshold (Ct) standard deviation (SD) fluctuations of 0.21–1.36, while TV-qPCR showed SD values of 0.58–2.79—representing 40–105% higher variability than the control system (Figure 5A). Similarly, for influenza A/B viruses, TV-qPCR SD values (0.50–1.48) exceeded those of control qPCR (0.14–0.98) (Figure 5B). These findings indicate opportunities for optimizing thermal cycling uniformity in TV-qPCR, particularly regarding temperature module stability during high-throughput testing.

Notably, TV-qPCR achieves significant functional advancements. As shown in Figure 5C, the system enables simultaneous multi-channel detection, performing qualitative and quantitative analysis of multiple targets (*ORF1ab*, *N*, *E*, *IFA*, *IFB*). The amplification curves demonstrate complete morphology with distinct exponential phases, confirming its integrated detection capability for complex applications including differential pathogen diagnosis and co-infection detection.

### 3.4. Comparative Performance Evaluation of POCT Devices Using LAMP Technology

As the gold standard in molecular diagnostics, qPCR technology demonstrates excellent laboratory performance, yet faces timeliness challenges during acute infectious disease outbreaks. This study innovatively integrates RT-LAMP with the TV-qPCR system to explore rapid detection optimization pathways.

**Figure 6:**
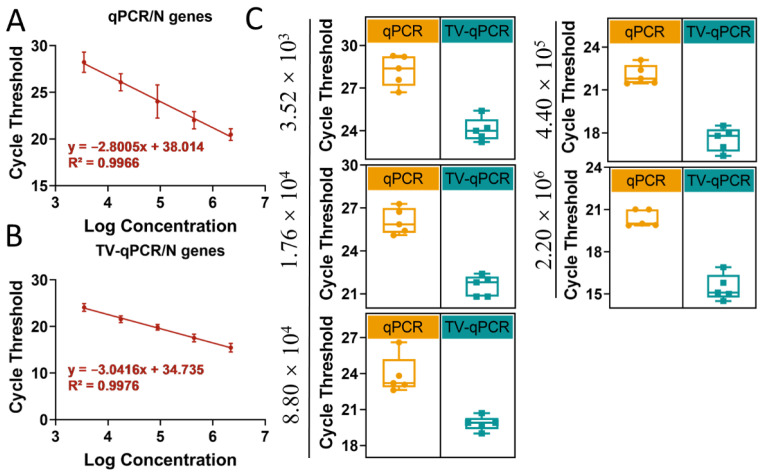
Stability and amplification curve testing of portable multifunctional nucleic acid detection devices (TV-qPCR) versus conventional fluorescence quantitative PCR (qPCR) equipment for reverse transcription loop-mediated isothermal amplification (RT-LAMP). (**A**), Linear range of qPCR device for novel coronavirus *N* gene. (**B**), Linear range of TV-qPCR device for novel coronavirus *N* gene. (**C**), Data stability of TV-qPCR device and qPCR device for novel coronavirus *N* gene.

Using SARS-CoV-2 *N* gene as the detection target, we conducted parallel RT-LAMP assays on TV-qPCR and conventional qPCR platforms for comparative analysis (Figure 6). Notably, the system employs standardized lyophilized reagent spheres—pre-encapsulating all buffer components except templates and primers—enabling rapid resolubilization that significantly enhances field-operation efficiency.

Data revealed highly consistent analytical performance between TV-qPCR and conventional systems in RT-LAMP detection: (1) Both covered identical linear ranges (3.52 × 10^3^–2.2 × 10^6^ copies/μL); (2) Standard curve regression coefficients (TV-qPCR R^2^ = 0.992; conventional R^2^ = 0.995) satisfied clinical requirements (Figure 6A,B); and (3) Cycle threshold standard deviation ranges (0.35–1.82 vs. 0.28–1.64, respectively) demonstrated comparable reproducibility (Figure 6C).

This performance convergence highlights TV-qPCR’s adaptability for isothermal amplification—its precision temperature control module maintains stringent thermal uniformity essential for LAMP technology.

To further evaluate the clinical performance, we tested 60 residual nasopharyngeal swab samples from suspected cases using the RT-LAMP mode on our portable device. The results showed the detection of 8 influenza A virus (IVA)-positive, 11 human rhinovirus (HRV)-positive, and 6 adenovirus (ADV)-positive samples, which were in complete agreement with the hospital’s standard test results (100% concordance rate; see Table S4). This demonstrates the reliable clinical utility of our portable device for rapid pathogen detection.

The TV-qPCR system demonstrated breakthrough advantages in RT-LAMP performance metrics (Table S5). Experimental data revealed TV-qPCR achieved a 23% lower detection limit (LOD: 2.95 copies/μL) than conventional qPCR (3.64 copies/μL). Its quantification limit (LOQ) showed even greater improvement—at 26.47 copies/μL, representing a 47% reduction versus the control system (50.09 copies/μL). This dual-low characteristic (low LOD + low LOQ) provides twofold advantages for highly transmissible pathogens: enabling earlier case detection and accurate low-viral-load quantification, thus supporting critical epidemic containment within the “golden 24-h” window.

Notably, TV-qPCR elevates RT-LAMP’s POCT suitability to unprecedented levels. While traditional RT-qPCR requires >2-h testing cycles, TV-qPCR completes the entire RT-LAMP workflow (sample lysis → nucleic acid amplification → result interpretation) in 42 min (Table S6). This efficiency breakthrough stems from unique engineering: (1) Microfluidic chips enable “single-button start” operation via pre-embedded lyophilized reagent chambers, reducing manual steps by 80%; and (2) Integrated thermostatic modules maintain precise temperature control (±0.2 °C at 65 °C), boosting amplification efficiency by 18% versus conventional equipment, This also suggests that integrating upstream sample preparation is a key objective for the future development of fully automated “sample-to-result” systems.

## 4. Discussion

Currently, as the gold standard for pathogen diagnosis, nucleic acid testing primarily relies on two technologies: RT-qPCR (highly sensitive but requiring bulky equipment) and RT-LAMP (rapid and portable but limited in multiplex detection capability). However, their functional fragmentation and compatibility constraints hinder flexible deployment in POCT scenarios, particularly for rapid tumor detection. This study achieves a breakthrough by developing a dual-functional device integrating both RT-qPCR and RT-LAMP methodologies, thereby bridging a critical gap in portable diagnostics. By leveraging the complementary strengths of both technologies, the device enables scenario-adaptive detection—providing unparalleled flexibility for diverse clinical and field applications while overcoming a key limitation of traditional instruments: impracticality in mobile laboratory settings [22,23]. Furthermore, Tesla valve optimization reduces structural complexity and enables controlled microchannel reflux. The compact design (13.5 × 7.5 × 10 cm) occupies just 10% of the volume of conventional qPCR systems. While published studies describe similarly sized devices, these typically support only RT-LAMP functionality [13,18,20]. Additionally, the device incorporates a four-channel optical detection system capable of simultaneously quantifying CY5, FAM, VIC, and ROX fluorophores—demonstrating unique utility in differential diagnosis of co-infections [24,25]. This high-level integration aligns with global trends in nucleic acid testing equipment development [26]. Research further indicates that devices with comparable channel counts typically exhibit larger footprints, lower detection efficiency, and higher LOD values [27].

This device’s performance metrics underscore its transformative potential for POCT. Comparative analysis with traditional qPCR systems reveals significant reductions in amplification time (27% faster for RT-qPCR, 14% faster for RT-LAMP) alongside superior analytical sensitivity for RT-LAMP (LOD: 2.95 copies/μL; LOQ: 26.47 copies/μL) and enhanced stability—outperforming most comparable devices reported in the literature [15,17,28]. This is primarily attributable to the fully sealed structure of the disposable chip, the low thermal capacity of the reaction chamber, and the integrated Tesla valve functionality. Collectively, these features minimize vapor escape and prevent backflow. Weight fluctuations in the chip before and after testing remained below 0.20 mg, while the temperature within the reaction chamber remained stable and uniform at each set point, with deviations within ±0.5 °C (Figure S4). Its modular architecture enables flexible mode selection: depending on the scenario, the operating program can be manually configured for either RT-PCR mode or RT-LAMP mode (see Section 3.2 in the Supplementary Materials). RT-qPCR mode serves high-sensitivity clinical confirmation needs, while RT-LAMP mode facilitates rapid outbreak screening with accelerated processing during emergencies versus conventional methods [29,30]. This efficiency gain critically narrows pandemic detection windows, with literature indicating each day saved through rapid diagnosis reduces secondary transmission risk by 20% [31]. However, due to instrument size constraints, only one sample can be loaded at a time, and the same sample cannot undergo both modes simultaneously or sequentially. Users may select either mode for testing based on circumstances. Portable deployment can increase remote area testing capacity by 60–80% [32], while cost-effectiveness analyses demonstrate that 90% cost reductions can yield 3–5× greater screening coverage [33]. The miniaturized design is projected to substantially lower procurement, operational, and maintenance expenses—a strategic imperative for implementing high-frequency viral surveillance in resource-limited regions. Concurrently, literature-proposed rapid response mechanisms may reduce public health emergency expenditures by >30% [31]. These combined attributes render the device exceptionally suited for resource-constrained environments, rapid outbreak response, and decentralized healthcare scenarios where portability, speed, and multiplex detection are paramount. Crucially, its technical alignment with World Health Organization (WHO)’s “diagnosis as treatment” strategy directly advances sustainable development goals for global health security [34].

Looking ahead, future research will focus on further optimizing RT-qPCR thermal cycling protocols and fluorescence signal stability. In this study, the stability of TV-PCR instrument in RT-PCR detection is inferior to that of traditional large-scale PCR instruments, while it shows comparable data stability to traditional large-scale PCR instruments in RT-LAMP detection. We believe that the larger heat capacity of traditional instruments is beneficial for maintaining temperature stability, while small devices are easily affected by environmental temperature fluctuations. RT-PCR requires rapid temperature cycling, which poses challenges to the heating/cooling design of small devices [35,36]. Meanwhile, in small devices, the signal of the photodetector may be interfered with due to space limitations. Especially for real-time PCR systems that require fluorescence quantitative detection, the impact of this optical noise will be greater [37,38]. In addition, the distribution of fluids within the chip may exert an even greater influence. PCR is highly sensitive to the spatial arrangement of reaction liquids [39], and compact microfluidic devices may encounter challenges arising from restricted or non-uniform reagent distribution. In contrast, LAMP operates under isothermal conditions and is therefore less affected by variations in fluid distribution. Based on these considerations, further improvements could include optimizing the temperature control system [40], refining the microfluidic design to achieve more uniform reagent distribution [39,41], and incorporating partitioned optical-path isolation to enhance the signal-to-noise ratio [38,42]. After that, the limitation of the current device lies in the absence of a heated lid, which results in slight evaporation of the liquid. However, the single-use, sealed miniaturized chips used in the experiments are designed with a simplified Tesla valve, which helps minimize liquid evaporation. Future improvements could involve reducing the size of the chip and incorporating a heated lid structure, further enhancing the system’s accuracy. Notably, the expansion of the device’s capabilities to enable cross-pathogen detection and the integration of AI-driven data interpretation with real-time epidemiological surveillance will enhance its application. This innovation not only bridges the gap between laboratory-grade accuracy and field-deployable diagnostics but also establishes a scalable platform for next-generation molecular testing. Clinical validation in various environmental conditions and diverse populations will be vital for its integration into global healthcare infrastructures.

## Data Availability

The dataset is not currently available for public sharing.

## Supplementary Materials

The following supporting information can be downloaded at: https://www.mdpi.com/article/10.3390/bios16010051/s1, Figure S1: Detailed drawing of the chip and vacuum chamber. (A) Detailed drawing of the Tesla valve chip and vacuum chamber, (B) Vacuum chamber opening and closing display; Figure S2: Power adapter power rating; Figure S3: Hangzhou BORI Technology Co., Ltd (FQD-96A) real-time fluorescence quantitative PCR instrument; Figure S4: PCR thermal cycling curve; Figure S5: ORF1ab, E, N genes amplification curve; Figure S6: IFA, IFB genes amplification curve; Figure S7: Comparison of fluorescence changes before and after different channel reactions; Figure S8: Control panel of the device; Figure S9: Device panel for configuring experimental settings; Figure S10: Historical experimental processes (left side); Figure S11: Experimental operation interface; Figure S12: Data analytics interface; Table S1: Target genes, primers and probes of qPCR; Table S2: LAMP primers for the Covid-19 N gene; Table S3: Time required for qPCR and TV-qPCR tests for RT-qPCR; Table S4: The limit of detection (LOD) and the limit of quantification (LOQ) values of N gene obtained by qPCR and TV-qPCR for RT-LAMP; Table S5: Time required for qPCR and TV-qPCR tests for RT-LAMP; Table S6: Research progress of POCT equipment in the direction of virus detection; Table S7: Comparison of experimental coefficients between TV-qPCR and qPCR; Table S8: Virus sample detection rate.
