# Supplementary material for: A Portable Dual-Mode Microfluidic Device Integrating RT-qPCR and RT-LAMP for Rapid Nucleic Acid Detection in Point-of-Care Testing
Source: Biosensors (Basel). 2026 Jan 8;16(1):51. doi: 10.3390/bios16010051 (PMC12839313; doi:10.3390/bios16010051)
Supplement: Supplementary file 1 [file biosensors-16-00051-s001.zip › biosensors-3989640-supplementary.pdf]

Supporting Information

Development and Performance Evaluation of a POCT Fluorescent Detection Device Integrating RT - PCR and RT - LAMP Detection Functions

Table of Contents

1. Supporting Figures 3
2. Supporting Tables..5
3 Application Scenarios and Workflow and Workflow......................................8
4. Supporting References..10

1. Supporting Figures

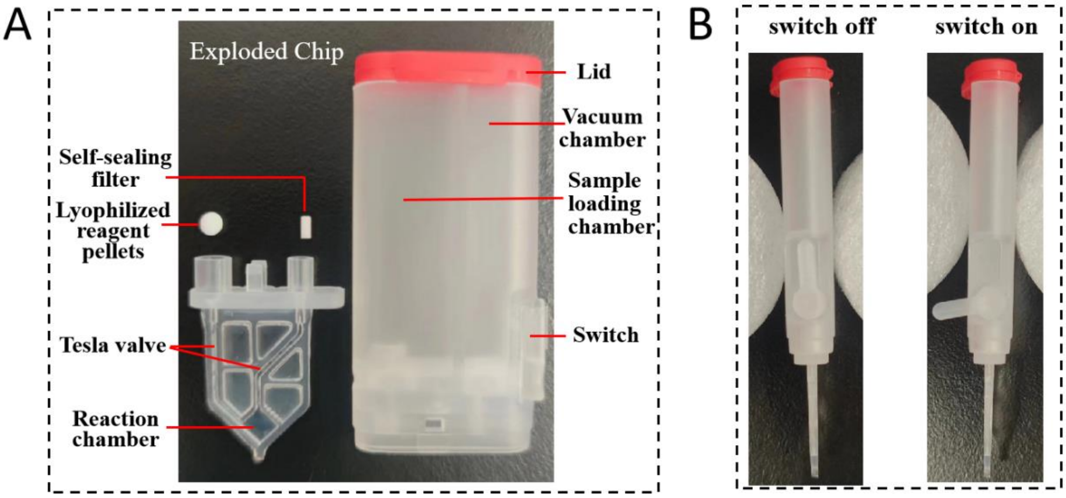

Figure S1. Detailed drawing of the chip and vacuum chamber. (A) Detailed drawing of the Tesla valve chip and vacuum chamber, (B) Vacuum chamber opening and closing display.

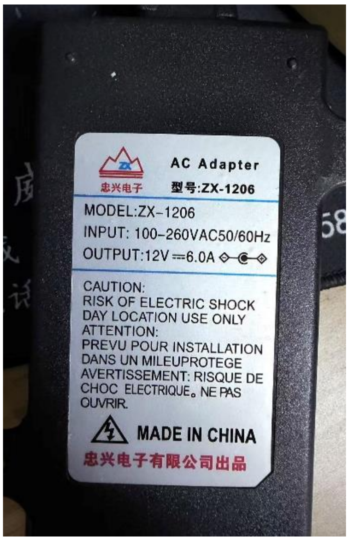

Figure S2. Power adapter power rating.

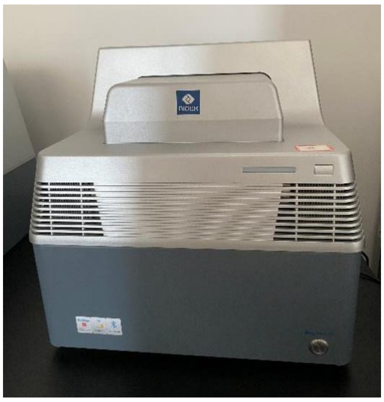

Figure S3. Hangzhou BORI Technology Co., Ltd (FQD-96A) real-time fluorescence quantitative PCR instrument.

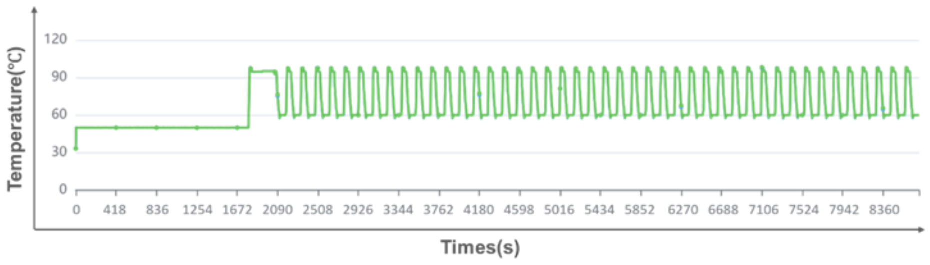

Figure S4. PCR thermal cycling curve.

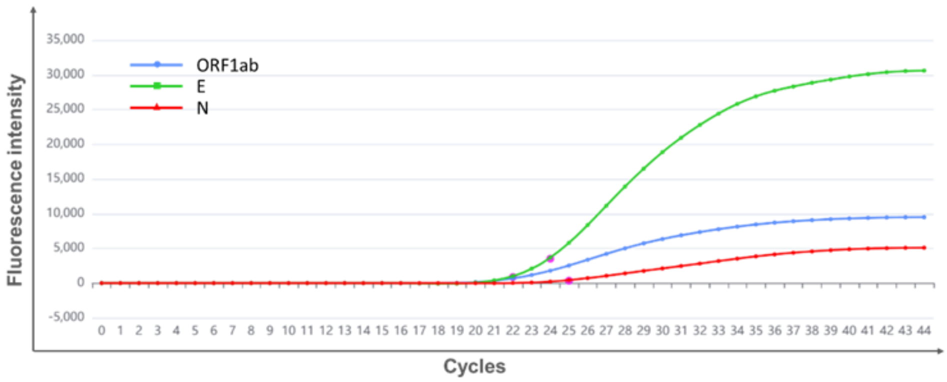

Figure S5. ORF1ab, E, N genes amplification curve.

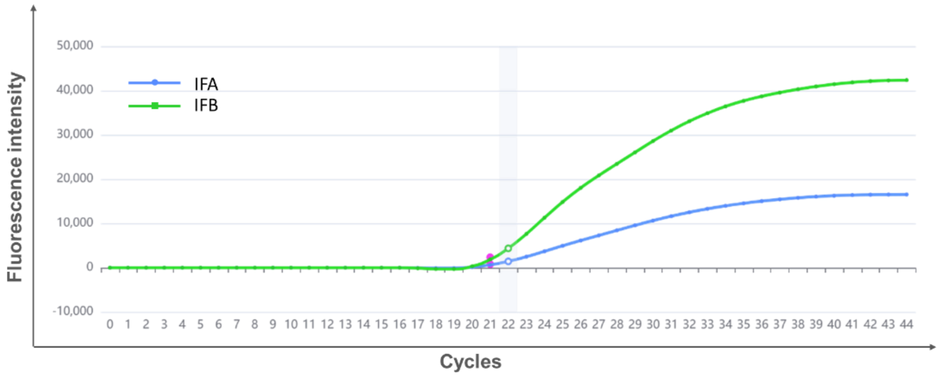

Figure S6. IFA, IFB genes amplification curve.

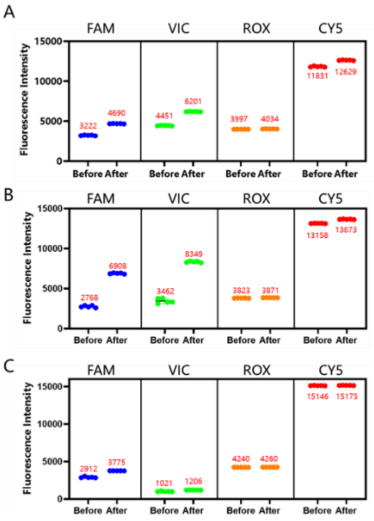

Figure S7. Comparison of fluorescence changes before and after different channel reactions.

2. Supporting Tables

Table S1. Target genes, primers and probes of qPCR

Target gene	Primer (5'–3')	Probe (5'–3')
ORF1a b	F: CCCTGTGGGTTTTACACTTAA<br>R: ACGATTGTGCATCAGCTGA	5'FAM–CCGTCTGCGGTATGTGGAAAGGTTATGG –3'BHQ1
N	F: GGGGAACTTCTCCTGCTAGAAT<br>R: CAGACATTTTGCTCTCAAGCTG	5'VIC–TTGCTGCTGCTTGACAGATT–3'TAMRA
E	F: GGAAGAGACAGGTACGTTAATA<br>R: AGCAGTACGCACACAATCGAA	5'CY5–ACACTAGCCATCCTTACTGCGCTTCG –3'BHQ2
IFA	F:CTTCTAACCACCGAGGTCGAAAC GTA<br>R:GGTGACAGGATTGGTCTTGTCTT TA	5'FAM–TCAGGCCCCCTCAAAGCCGAG–3'BHQ1
IFB	F:TACGGTGGATTAAACAAAAGC<br>R: TGCAGGAGGTCTATATTTGGT	5'VIC–AGCCTTACTACACAGGAGAACATGC–3'T AMRA

Table S2. LAMP primers for the Covid-19 N gene

Name	Primer (5'–3')
N-F3	AGATCACATTGGCACCCG
N-B3	CCATTGCCAGCCATTCTAGC
N-FIP	TGCTCCCTTCTGCGTAGAAGCCAATGCTGCAATCGTGCTAC
N-BIP	GGCGGCAGTCAAGCCTCTTCCCTACTGCTGCCTGGAGTT
N-LF	GCAATGTTGTTCCTTGAGGAAGTT
N-LB	GTTCCTCATCACGTAGTCGCAACA

Table S3. Time required for qPCR and TV-qPCR tests for RT-qPCR.

	qPCR	TV-qPCR
Time (h)	1.79 ± 0.03	1.32 ± 0.04

Table S4. The limit of detection (LOD) and the limit of quantification (LOQ) values of N gene obtained by qPCR and TV-qPCR for RT-LAMP

	LOD (copies/μL)		LOQ (copies/μL)	
	qPCR	TV-qPCR	qPCR	TV-qPCR
N gene	3.64	2.95	50.09	26.47

Table S5. Time required for qPCR and TV-qPCR tests for RT-LAMP

	qPCR	TV-qPCR
Time (min)	48.27 ± 0.35	41.79 ± 0.13

Table S6. Research progress of POCT equipment in the direction of virus detection

Study	Detection	LOD （copies/μl）	Efficiency /min	Volume (L×W×H/cm）	Fluorescence Channel
Jayanath et al. [1]	RT-LAMP		60		
Hu et al.[2]	RT-LAMP		60	9/9/10	
Chen et al. [3]	RT-LAMP	20	80		
Mohit et al. [4]	RT-LAMP	200	60	6mm×20mm	
Kang et al.[5]	Nano plasma PCR	9.55×10^4	5	14 mm × 26 mm × 4mm	One
Nguyen et al. [6]	RT-LAMP	20	33	5.5/9/10	Four
Hoan et al [7]	RT-PCR	0.5	15		
Liu et al [8]	RT-LAMP	16	40	8.8/6.8/8.2	Three
Zhang et al [9]	digital PCR	20.24	52	29/25/48	One
Xu et al [10]	digital PCR	$3.0 \times 10^1 \sim 1.5 \times 10^5$	120	20/15/24	FAM/VIC/Cy3/Cy5
Renzoni et al [11]	RT-PCR	2.1×10^3	30		

Table S7. Comparison of experimental coefficients between TV-qPCR and qPCR

	RT-qPCR						
	Sensitivity (copies/μl)	LOD/LOQ (copies/μl)	Stability	Fluorescence Channel	Efficiency （min）	Volume （L×W×H/cm）	Weight (kg)
qPCR	10^1-10^5	1.79/5.87	0.21 ~ 1.36	6	107.4 ± 0.03	41×38.6×35.2	15
TV-qPCR	10^1/10^2-10^5	2.00/8.21	0.58 ~ 2.79	4	79.2 ± 0.04	13.5 × 7.5 × 10	0.8
	RT-LAMP						
	Sensitivity (copies/μl)	LOD (copies/μl)	Stability	Fluorescence Channel	Efficiency （min）	Volume （L×W×H/cm）	Weight (kg)
qPCR	10^3-10^6	3.64/50.09	0.35-1.82	6	48.27 ± 0.35	41×38.6×35.2	15

TV-qPCR	10^3-10^6	2.95/26.47	0.28-1.64	4	41.79 ± 0.13	13.5 × 7.5 × 10	0.8

Table S8. Virus sample detection rate

Virus type	IVA	HRV	ADV
**Detected ratio**	**8/8**	**11/11**	**6/6**
**Detection accuracy rate**	**100%**	**100%**	**100%**

3 Application Scenarios and Workflow and Workflow

3.1. Network Connection

After connecting the computer to the TV-PCR device's dedicated Wi-Fi network, open the control panel. The display interface appears as follows:

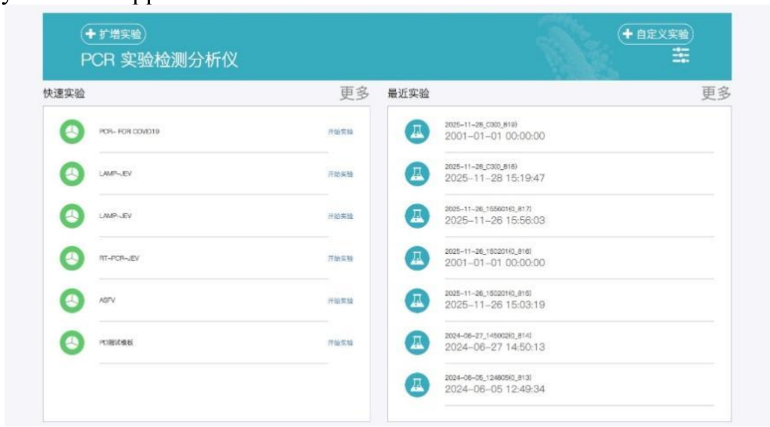

Figure S8. Control panel of the device.

3.2. Programme Settings

Select the custom mode on the right to set the name for each experiment. Choose the appropriate temperature programme according to the selected mode. Select the corresponding fluorescence channel based on the sample's reaction fluorescence; multiple channels may be selected for simultaneous detection at this stage.

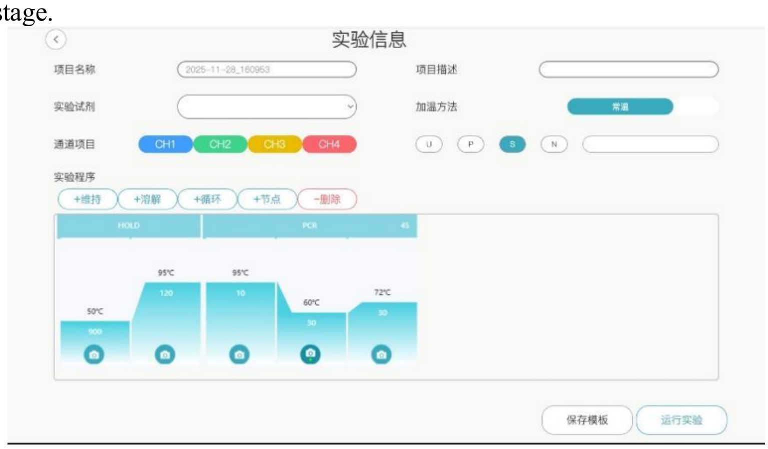

Figure S9. Device panel for configuring experimental settings.

3.3. Template Storage

Upon completing the configuration of RT-PCR and RT-LAMP protocols respectively, save them as experimental templates. The established experimental procedures will be stored on the left side of the control panel. Subsequent experiments may select saved rapid experimental protocols to commence detection directly.

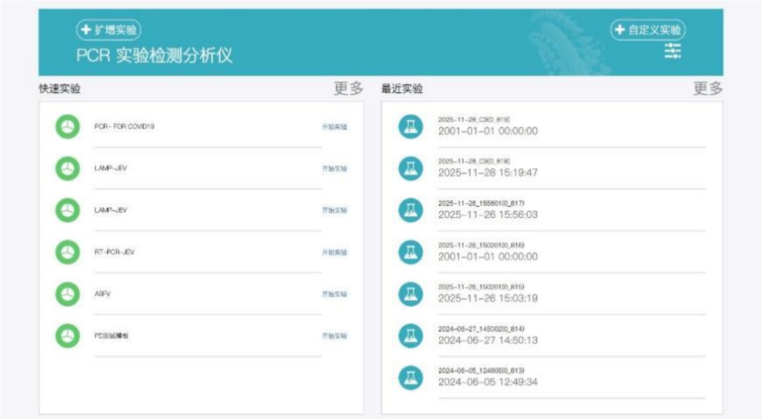

Figure S10. Historical experimental processes (left side).

3.4. Sample Loading

Transfer the samples to be amplified into the disposable chips, then insert the chips into the loading area.

3.5. Operation

During machine operation, the control panel displays real-time fluorescence, temperature, information, and progress, whilst recording the duration of the experiment.

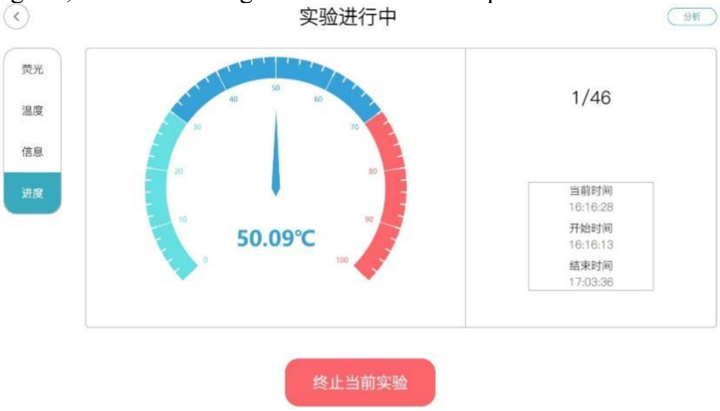

Figure S11. Experimental operation interface.

3.6. Data Analysis

Upon completion of the test, the instrument will automatically generate a test report. Experimental data may be saved directly or viewed within the panel interface.

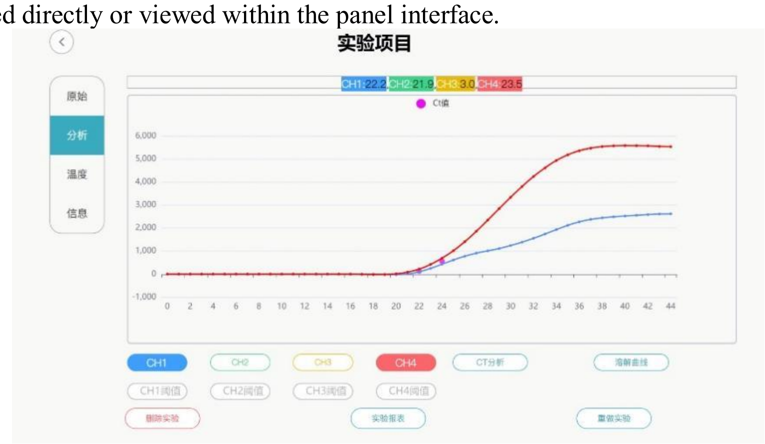

Figure S12. Data analytics interface.

4. Supporting References

[1] N.Y. Jayanath, L.T. Nguyen, T.T. Vu, L.D. Tran, Development of a portable electrochemical loop mediated isothermal amplification (LAMP) device for detection of hepatitis B virus, RSC Advances 8 (2018) 34954-34959. 10.1039/c8ra07235c.
[2] F. Hu, J. Li, Z. Zhang, M. Li, S. Zhao, Z. Li, N. Peng, Smartphone-Based Droplet Digital LAMP Device with Rapid Nucleic Acid Isolation for Highly Sensitive Point-of-Care Detection, Analytical Chemistry 92 (2019) 2258-2265. 10.1021/acs.analchem.9b04967.
[3] X. Chen, Q. Zhou, S. Li, H. Yan, B. Chang, Y. Wang, S. Dong, Rapid and Visual Detection of SARS-CoV-2 Using Multiplex Reverse Transcription Loop-Mediated Isothermal Amplification Linked With Gold Nanoparticle-Based Lateral Flow Biosensor, Frontiers in Cellular and Infection Microbiology 11 (2021). 10.3389/fcimb.2021.581239.
[4] J.L. Davidson, J. Wang, M.K. Maruthamuthu, A. Dextre, A. Pascual-Garrigos, S. Mohan, S.V.S. Putikam, F.O.I. Osman, D. McChesney, J. Seville, M.S. Verma, A paper-based colorimetric molecular test for SARS-CoV-2 in saliva, Biosensors and Bioelectronics: X 9 (2021). 10.1016/j.biosx.2021.100076.
[5] B.-H. Kang, Y. Lee, E.-S. Yu, H. Na, M. Kang, H.J. Huh, K.-H. Jeong, Ultrafast and Real-Time Nanoplasmonic On-Chip Polymerase Chain Reaction for Rapid and Quantitative Molecular Diagnostics, ACS Nano 15 (2021) 10194-10202. 10.1021/acsnano.1c02154.
[6] H.Q. Nguyen, H.K. Bui, V.M. Phan, T.S. Seo, An internet of things-based point-of-care device for direct reverse-transcription-loop mediated isothermal amplification to identify SARS-CoV-2, Biosensors and Bioelectronics 195 (2022). 10.1016/j.bios.2021.113655.
[7] H.T. Ngo, M. Jin, A.Y. Trick, F.-E. Chen, L. Chen, K. Hsieh, T.-H. Wang, Sensitive and Quantitative Point-of-Care HIV Viral Load Quantification from Blood Using a Power-Free Plasma Separation and Portable Magnetofluidic Polymerase Chain Reaction Instrument, Analytical Chemistry (2022). 10.1021/acs.analchem.2c03897.
[8] T. Liu, A.J. Politza, A. Kshirsagar, Y. Zhu, W. Guan, Compact Point-of-Care Device for Self-Administered HIV Viral Load Tests from Whole Blood, ACS Sensors 8 (2023) 4716-4727. 10.1021/acssensors.3c01819.
[9] W. Zhang, L. Cui, Y. Wang, Z. Xie, Y. Wei, S. Zhu, M. Nawaz, W.-C. Mak, H.-P. Ho, D. Gu, S. Zeng, An Integrated ddPCR Lab-on-a-Disc Device for Rapid Screening of Infectious Diseases, Biosensors 14 (2023). 10.3390/bios14010002.
[10] L. Xu, H. Qu, D.G. Alonso, Z. Yu, Y. Yu, Y. Shi, C. Hu, T. Zhu, N. Wu, F. Shen, Portable integrated digital PCR system for the point-of-care quantification of BK virus from urine samples, Biosensors and Bioelectronics 175 (2021). 10.1016/j.bios.2020.112908.
[11] A. Renzoni, F. Perez, M.T. Ngo Nsoga, S. Yerly, E. Boehm, A. Gayet-Ageron, L. Kaiser, M. Schibler, Analytical Evaluation of Visby Medical RT-PCR Portable Device for Rapid Detection of SARS-CoV-2, Diagnostics 11 (2021). 10.3390/diagnostics11050813.
